# Multiview deconvolution approximation multiphoton microscopy of tissues and zebrafish larvae

**DOI:** 10.1038/s41598-021-89566-w

**Published:** 2021-05-12

**Authors:** Dimitrios Kapsokalyvas, Rodrigo Rosas, Rob W. A. Janssen, Jo M. Vanoevelen, Miranda Nabben, Martin Strauch, Dorit Merhof, Marc A. M. J. van Zandvoort

**Affiliations:** 1 Department of Genetics and Cell Biology, Faculty of Health, Medicine and Life Sciences (FHML), Maastricht University, Maastricht, the Netherlands; 2 Institute for Molecular Cardiovascular Research (IMCAR), University Hospital RWTH Aachen University, Aachen, Germany; 3 Institute of Imaging and Computer Vision, RWTH Aachen University, Aachen, Germany

**Keywords:** Microscopy, Transgenic organisms, 3-D reconstruction, Fluorescence imaging, Microscopy, Multiphoton microscopy

## Abstract

Imaging in three dimensions is necessary for thick tissues and small organisms. This is possible with tomographic optical microscopy techniques such as confocal, multiphoton and light sheet microscopy. All these techniques suffer from anisotropic resolution and limited penetration depth. In the past, Multiview microscopy—imaging the sample from different angles followed by 3D image reconstruction—was developed to address this issue for light sheet microscopy based on fluorescence signal. In this study we applied this methodology to accomplish Multiview imaging with multiphoton microscopy based on fluorescence and additionally second harmonic signal from myosin and collagen. It was shown that isotropic resolution was achieved, the entirety of the sample was visualized, and interference artifacts were suppressed allowing clear visualization of collagen fibrils and myofibrils. This method can be applied to any scanning microscopy technique without microscope modifications. It can be used for imaging tissue and whole mount small organisms such as heart tissue, and zebrafish larva in 3D, label-free or stained, with at least threefold axial resolution improvement which can be significant for the accurate quantification of small 3D structures.

## Introduction

In the last two decades, numerous novel optical microscopy imaging methods have been developed. They centre around increasing resolution below the diffraction limit (Super resolution methods), increasing penetration depth in tissue (Multiphoton microscopy and tissue clearing), and increasing speed and reducing photodamage for high-throughput imaging of large fields of view (Light sheet microscopy). Whatever their specific goal, all optical microscopy methods suffer from anisotropic resolution. Consequently, the details visible in the axial plane (XZ or YZ plane) are of inferior quality to those visualized in the imaging plane (XY-plane). When imaging flat samples such as cultured cells, the manifestation of this anisotropic resolution may not be as significant, however when imaging thick samples, such as tissues or small organisms, it will result in blurry 3D objects. This is more pronounced for multiphoton microscopy (MPM), where typically water immersion objectives between 0.6–1.0 NA are used. Based on the theoretical resolution of MPM^1^, for an 1.0 NA objective at 820 nm excitation, anisotropy (the ratio of lateral to a resolution) is 0.27, whereas for a 0.6 NA objective it is 0.13. Consequently, objects with size in the range of the excitation wavelength, will appear very elongated in the axial dimension and this distortion is exacerbated with lower NA objectives.

Anisotropic resolution in microscopy has been addressed before. It has been shown that the use of two opposing objectives creates an interference excitation field, which significantly reduces the axial size of the point spread function (PSF) and therefore axial resolution. This was implemented in scanning mode through 4Pi microscopy^2^ and in widefield mode in I^5^M ^3^. Both techniques required deconvolution to remove shadow effects and were able to produce images with 100 nm axial resolution. However there were limitations in the sample thickness (some μm), efficient removal of artifacts, and alignment of the opposing beams^4^, which limited their applicability and wide spreading to the biomedical community. Another method proposed was the tilted view imaging^5^ of the sample and subsequent reconstruction of the 3D image. Although proposed almost three decades ago it did not find many applications due to difficulties in implementation and image processing.

The idea of tilted view^5^ or multiview imaging was later adapted in Light Sheet Fluorescence Microscopy^6^ (LSFM) (or Selective Plane Illumination microscopy – SPIM), to decrease anisotropy in resolution. Implementation of this so-called Multiview Imaging (MVI) in LSFM, meaning imaging the sample from different angles, allowed achievement of isotropic resolution (anisotropy equals 1)^7,8^. Since detection is camera based on a widefield configuration, LSFM is a fast technique with high signal-to-noise ratio (SNR). Using LSFM has enabled imaging and tracking of single cells, in vivo imaging of the development of whole embryos of drosophila, *Caenorhabditis elegans*, and zebrafish, and imaging of large fixed and cleared specimens such as whole brain of rodents at high speed^9–14^. MVI has become a standard imaging technique in LSFM, and it can be either applied sequentially or simultaneously in order to reduce acquisition time^11,14,15^. Further increase of resolution in MVI can be accomplished through deconvolution^8,14^. By applying MVI deconvolution (MVD) the artifacts caused by the elongated PSF of each view can be minimized and this can improve further the contrast and decrease the resolution of the image. This is particularly useful when the size of the imaged structures are in the range of the excitation wavelength^8,14^.

Despite computational power being readily available, MVI, has not found many applications in scanning microscopy, such as MPM and confocal microscopy. Indeed, some applications in MPM have been demonstrated, based on the signal of Second Harmonic Generation (SHG) from collagen fibres^16,17^. However, MVD has not been demonstrated. This additional step could offer improved contrast and decreased resolution.

MPM has the advantages of deeper tissue penetration, due to the use of near infrared excitation, lower photobleaching, due to the exclusive focal excitation, excitation of multiple fluorophores with a single excitation wavelength, due to increase excitation pathways^18^. Moreover it has the unique advantage of inducing Second Harmonic Generation (SHG) signal in the proteins collagen, myosin, and tubulin^19^. These advantages allow imaging thick tissue sections^20^ or small organisms^21^, detection of multiple signals simultaneously^22^, imaging of stained structures but also detection of inherent autofluorescence signal and SHG ^23^, with reduced overall photobleaching.

In this study the possibilities and capabilities of applying MVD in MPM, based on both fluorescence and SHG contrast were explored. For this purpose, a custom rotation chamber was constructed and was used on a commercial MPM. Image processing was performed with processing tools already developed for LSFM. MVD was performed on fluorescence and MVD approximation (MVDA) on SHG signal. It was demonstrated that isotropic resolution could be achieved with MVD and that this method could be applied to any scanning microscope without microscope modifications.

## Materials and Methods

### Samples

Zebrafish larvae at 3 days post fertilization (dpf) were used. The transgenic line sensory:GFP was used^24^ to visualize all sensory neurons in the developing larvae in a nacre background, lacking melanophores^25^, thus eliminating interference from pigmented cells. Larvae were fixed for 15 min in 4% formaldehyde and stored in PBS at 4 °C until further use. Subsequently, nuclei were stained using SYTOX orange 2 nM (Invitrogen, LOT: 1933342) for 10 min. For heart tissue imaging, the heart from a zebrafish larva was dissected after fixation. Rat-tail tendon was extracted from a healthy adult rat, fixated in 4% formaldehyde for 15 min, and stored in PBS. For imaging, a small section was cut and used. All samples were stored at 4 °C. The experiments in this study were performed according to Dutch regulations and approved by the Dutch Central Committee of Animal Use (CCD) and the Maastricht University Committee for Animal Welfare. A specific animal study protocol for the zebrafish was not required since the samples used were younger than 5dpf. Rat tail tendon was acquired based on the *Three Rs* principle from an approved CCD protocol (2016-004).

### PSF generation

Theoretical PSF were generated on *Huygens* (SVI, Hilversum, the Netherlands). Parameters used were 1.00 NA objective, water immersion, 820 nm excitation, 520 nm emission, excitation photons: 2, pinhole size 2500 nm.

### Imaging

For imaging, a Leica TCS SP5 (Leica Microsystems GmbH, Wetzlar, Germany) MPM was used, with a Ti–Sapphire Chameleon Ultra II (Coherent Inc, Santa Clara, CA, USA) laser. Excitation was at 820 nm. A Leica objective, HCX APO L 20x/1.00 was used. Fluorescence detection was performed using the de-scanned detectors set according to the emission spectra of the dyes used in each sample. Image acquisition was performed simultaneously for all channels. SYTOX was detected at 560–600 nm, GFP at 500–540 nm, and SHG signal from collagen and myosin were detected with a forward detector with a bandpass filter (380–420 nm). Fluorescence beads (FluoSpheres, Life Technologies Inc, Eugene, OR, USA, LOT: 1835801, excitation/emission 540/560 nm) were used as fiducial marker and detected in the same channel as SYTOX. Autofluorescence in the zebrafish sample was detected in the same channel as GFP.

### Rotation chamber

**Figure 1:**
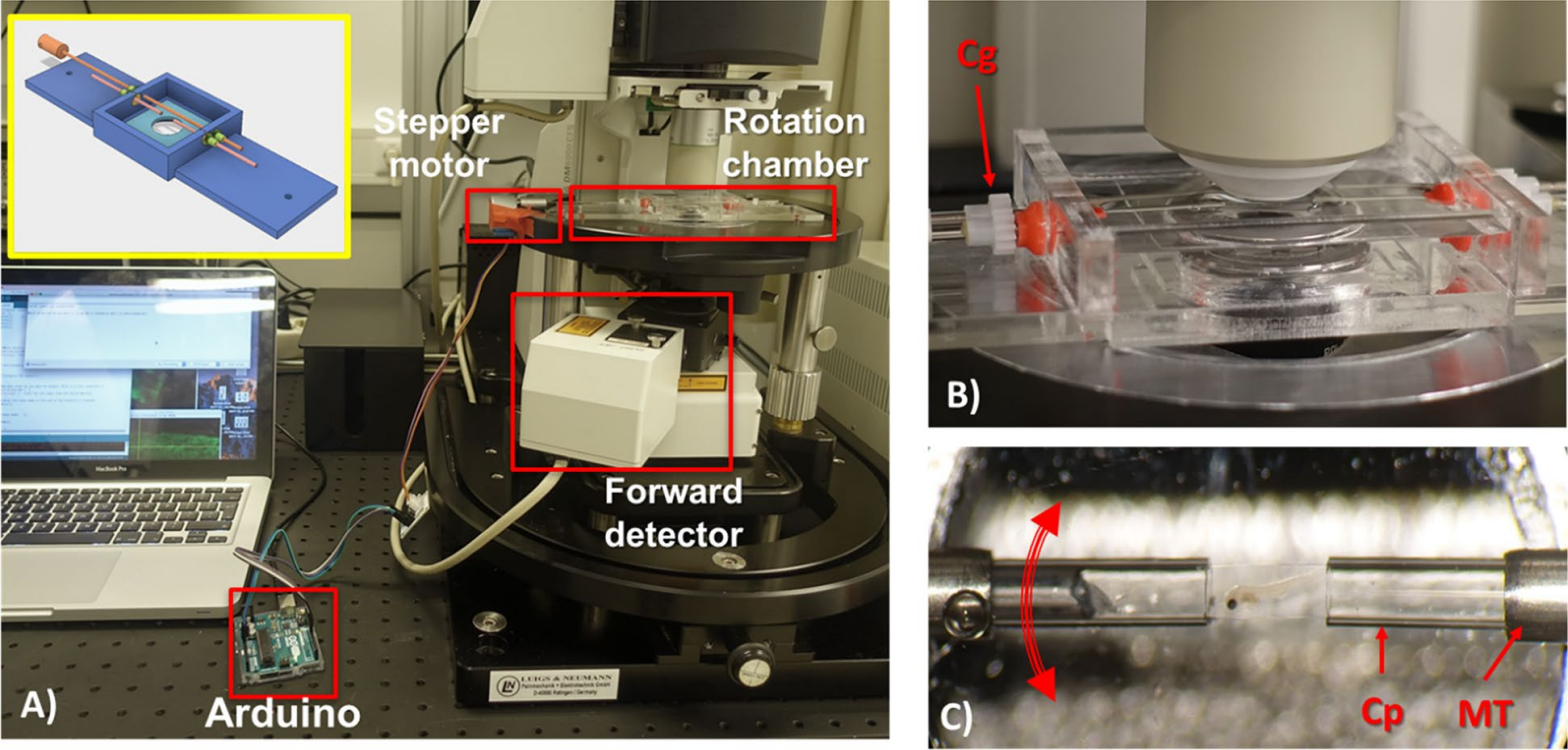
Rotation chamber for Multiview imaging with a two-photon microscope. (**A**) The rotation chamber mounted on the microscope stage is indicated. The stepper motor is attached to the shaft of the rotation chamber and is controlled by the Arduino board. On the inset of (**A**) the CAD design of the rotation chamber is shown. (**B**) The objective is focused on the sample in the rotation chamber. There is ample space for the objective to reach the sample. Below the chamber the condenser lens is positioned. The two cogs responsible for the rotation of the sample are indicated with Cg. (**C**) An image of the sample when it is ready for imaging. The sample is embedded in the agarose column and is extruded from the capillary allowing the objective to image the sample through the agarose layer. Red arrow indicates rotation direction. MT indicates the metallic tube, Cp indicates the haematocrit glass capillaries.

A custom chamber made of acrylic glass, was constructed (Fig. 1A). A motor, controlled by an Arduino board, was attached to the rotation shaft of the chamber for the rotation of the sample. The rotation shaft acted on two cogs on both side of the chamber. The two metallic tubes were rotated simultaneously by the rotation shaft. Inside the metallic tubes the glass capillary (Micro Haematocrit Tubes, d:1.55 μm, Vitrex, Herlev, Denmark) containing the sample were inserted. The capillaries were coupled to the rotating tubes with a silicon tube at the edges. The capillaries fit tightly in the tubes, so there is no wobbling during rotation. The chamber was mounted on the microscope stage between the objective and the condenser lens (NA:0.9) (Fig. 1B). The chamber was filled with d-water. Experiments were performed at room temperature (~ 21 °C).

### Sample preparation

The samples were immersed in 1% low melting point agarose gel (Sigma, LOT:SLBW8410) containing fluorescent beads. A stock solution of beads was prepared (2 μl dispersed in 0.5 ml water) and 16 μM of the stock solution was dissolved in 0.2 ml, 1% low melting point agarose containing the sample. The mixture, while still liquid, was suctioned in a haematocrit capillary. The capillary was subsequently mounted on the rotation chamber. After the mixture solidified (typically 1–2 min), it was extruded to the opposing capillary. The agarose column containing the sample was exposed to the objective (Fig. 1C). A drop of agarose on each side of the capillary was added to ensure the agarose column, containing the sample, was firmly attached to the rotating capillary.

Performing MVD includes several steps and caution must be taken to avoid pitfalls. Regarding sample mounting it is important to achieve good refractive index matching. For this reason, low melting point agarose was used, which has a refractive index similar to that of water (RI_agarose_:1.334, RI_water_:1.333). RI was measured with a refractometer (OPTi, Bellingham + Stanley, Kent, UK). The low agarose content makes the exposed agarose column fragile and caution must be taken on the length of this column so that it does not deform during rotation. In practice, exposed agarose columns of 3–4 mm where rigid enough (Fig. 1C).

### Sample rotation

The sample was rotated at discrete angles with a stepper motor controlled by an Arduino board. Sample could be rotated with 1° angle resolution and full 360° was possible. Rotation steps between 45°, and 90° angles, depending on the experiment were selected. Rotation is performed around the the long axis of the chamber. Rotation direction is indicated in Fig. 1C.

### Multiview image reconstruction and deconvolution

After acquisition, images were analysed with *Fiji*^26^. Registration, fusion, and deconvolution were performed with the Fiji plugin Multiview reconstruction^8^. Registration was based on the beads, which served as fiducial markers, embedded on the agarose column containing the sample. MVD was performed with the same plugin using the PSF extracted from the beads, with deconvolution setting “independent”, on 10 iterations.

## Results

### Multiview imaging resolution

**Figure 2:**
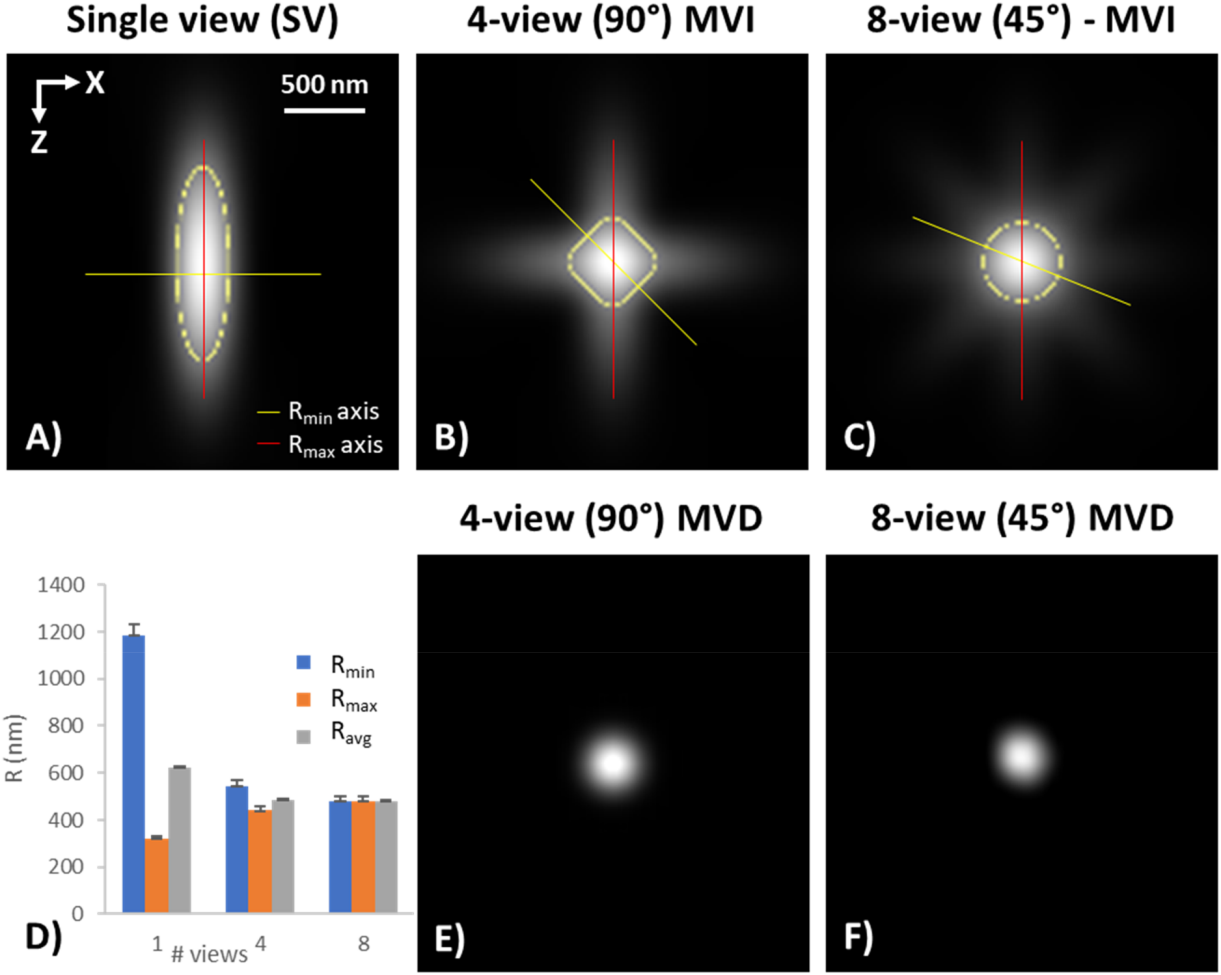
Multiview resolution. (**A**–**C**) Cross section of the PSF of (**A**) Single View (SV), (**B**) 4-views MVI, and (**C**) 8-views MVI. The yellow line indicates the axis of R_min_, the red line the axis of R_max_, and the yellow dotted line the area of the FWHM from which R_avg_ is extracted. (**D**) Graph of the R_min_, R_max_ and R_avg_ of the PSFs in (**A**–**C**). (**E**) 4-view deconvolved PSF of 2B. **F**) 8-view deconvolved PSF of 2C. (Images were created with Fiji^26^).

In this section, the PSF of single view (SV) and MVI were calculated. The focus of this study was on imaging thicker samples were all details are not equally visualized in all views, therefore multiple views are necessary to capture the whole object. We calculated the PSF for the ideal case where the PSF is not deformed due to aberrations at different depths and all details are equally visible in all views. The purpose of this calculation is to estimate the degree of resolution improvement and isotropy it can be achieved. Therefore, the PSF of SV, 4-view MVI, and 8-view MVI were calculated and compared. The theoretical PSF of an SV was generated with the *Huygens* software. It was calculated based on the parameters of a typical MPM objective of NA:1.0, water immersion, at 820 nm excitation. The MVI PSF was calculated by rotating the PSF of the SV with steps of 90° for the 4-view MVI and 45° for the 8-view MVI. The individual rotated PSFs of each view were added to create the MVI PSF for 4- and 8-view MVI. The resulting PSFs can be seen in Fig. 2. The MVI PSF was further deconvolved to produce the corresponding MVD.

The PSF of the SV (Fig. 2a) appears as an ellipsoid. The minimum resolution of a SV is found in the lateral (XY) axis and is 320 ± 13 nm, while the maximum in the Z axis (XZ or YZ) is 1183 ± 49 nm, in good agreement with the theoretical estimation^1^. The yellow dotted line in Fig. 2A indicates the Full width at half maximum (FWHM) in all directions around the centre. The average resolution was calculated as the effective diameter of the area formed by the FWHM, and it was found to be 624 ± 6 nm. Similar calculations were made for the 4-view MVI PSF in Fig. 2b. The PSF is maximum on the same axis as in the SV and was found to be 545 ± 23 nm, which is significantly smaller compared to that in SV. The axis where the PSF becomes minimum (R_min_) is located at 45°, in reference to the axis of maximum diameter of the PSF(R_max_), and it was 440 ± 18 nm; slightly bigger compared to that of the SV. The average PSF diameter based on the FWHM (R_avg_) was 485 ± 5 nm which is smaller compared to that observed in the SV. For 8-view MVI (Fig. 2c) the maximum PSF diameter was 480 ± 20 nm, minimum 480 ± 20 nm, and average 480 ± 5 nm. Anisotropy in resolution, defined as the ratio of lateral to axial resolution or equivalently as the ratio of R_min_ over R_max_, is 0.27 for SV, 0.80 for 4-view, and 1.00 for 8-view, thus in 8-view MVI configuration, isotropic resolution is achieved.

The MVI PSFs were deconvolved using the *Multiview Reconstruction*^8^ plugin of *Fiji*^26^ and the SV PSF. The resulting deconvolved PSFs showed higher resolution with R_min_: 230 ± 10 nm, R_max_: 0.26 ± 10 nm for 4-view, and R_min_ : 240 ± 10 nm R_max_: 240 ± 10 nm for 8-view MVD. Anisotropy is further improved with anisotropy of 0.88 for 4-views and 1.00 for 8-view. The corresponding deconvolved PSFs are presented in Fig. 2E and F.

Comparing the average MVI resolution of 8-, and 4-views to the SV, it becomes evident that MVI produces PSFs with smaller average dimensions, therefore offering better overall resolution. This is accomplished because the resolution is more isotropic compared to that in SV. The maximum and minimum PSF diameter tend to equalize with higher view number. While the absolute minimum in resolution can be achieved with SV in the XY plane, MVI will improve the average resolution. The 8-view MVI does offer the best average resolution (480 nm), however the improvement compared to 4-view MVI is very small. Additionally, deconvolution produces even smaller and very isotropic deconvolved PSFs (4-view R_avg_:0.245 nm, 8-view R_avg_:0.24 nm). Considering that the 8-view requires twice the acquisition time compared with 4-view, it was concluded that often 4-view MVI and MVD can offer a good compromise between speed and average resolution. Whether that is enough depends on the sample and how visible are all the details of the sample in all views.

### Multiview deconvolution with 4 views

A nacre zebrafish, expressing GFP on the pain sensory neurons, was used for 4-view MVD. The entire field of view (738 × 738 μm^2^) of the 20×, NA: 1.0 objective with a voxel size of 721 × 721 × 1482 nm^3^ was used. To decrease acquisition time a 1024 × 1024-pixel format was used, although formats up to 8192 pixels were possible. Four views at 90° rotation step were acquired with a total acquisition time of 25 min. Rotation axis was on the x-axis as indicated in Fig. 4. Excitation was at 820 nm and all three channels were acquired simultaneously. In the blue channel, SHG from muscle fibres (from the body), and collagen (mainly from the fins) were recorded. In the green channel, the signal of GFP was detected, as also a significant amount of autofluorescence. In the red channel, nuclei stained with SYTOX orange and the fluorescent beads, which were used as fiducial markers, were visualized. The nuclear stain did not penetrate in the body so only cells on the skin were stained, which gave a clear view of the larva’s external boundaries.

**Figure 3:**
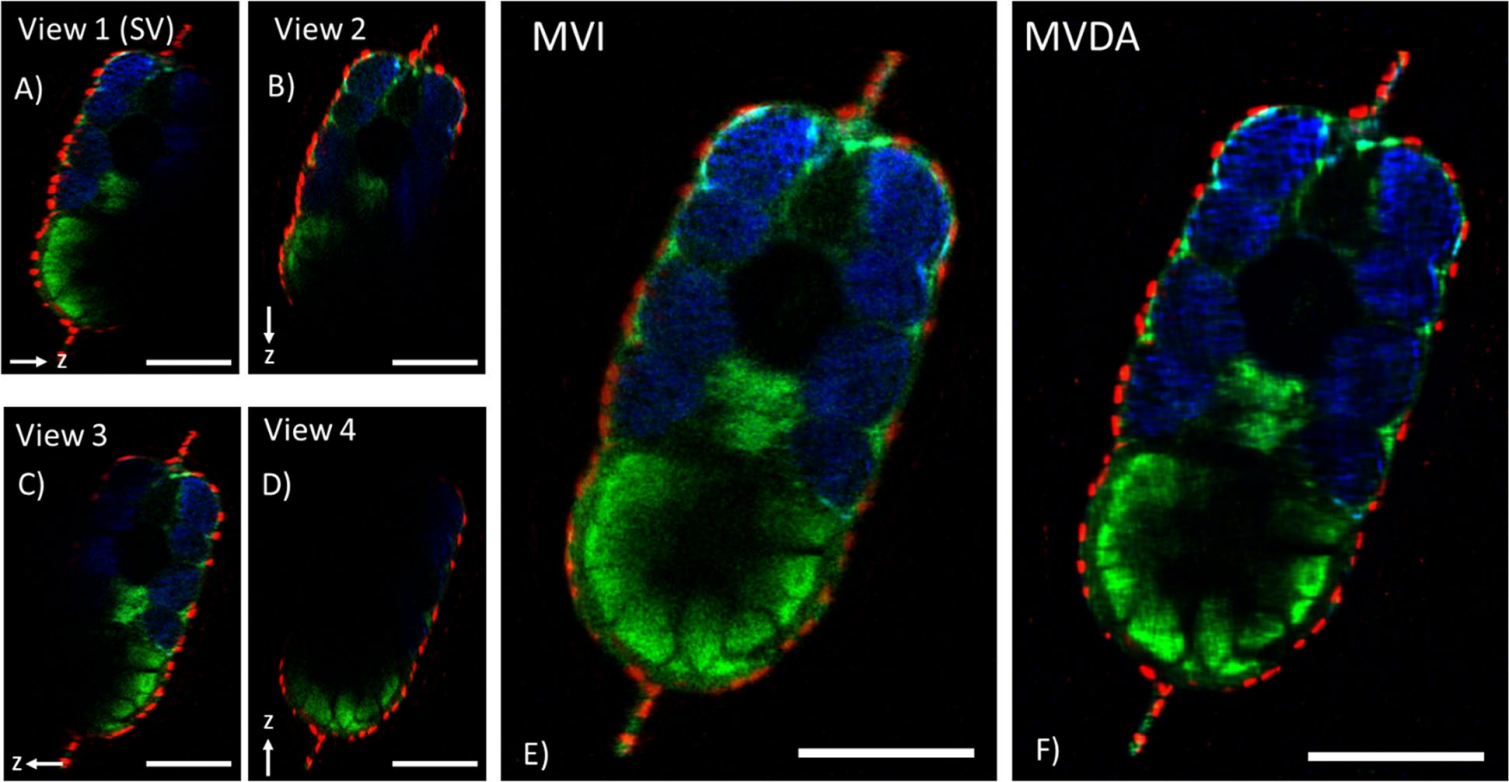
Cross section of the four view Multiview images of a 3dpf zebrafish sample. (**A**–**D**) Registered individual single view images. Arrow indicates the direction of the z axis of each view. Rotation axis for MVI was on the x-axis which is perpendicular to these images. (**E**) MVI (fusion) of single images, where the entirety of the sample is visible. (**F**) MV deconvolution approximation (MVDA) image. Structures appear with better contrast as noise is decreased and resolution improved. Low signal features become better visible in this image. Scale bars 100 μm. (Images were created with Fiji^26^).

In Fig. 3 the cross sections of the individual registered views, the fusion of the registered views (MVI), and the MVD approximation (MVDA) result are presented. Since the same PSF for deconvolution of the SHG signal was used, the term approximation is added in MVD to differentiate it from classical deconvolution. First step in the analysis is the registration of each individual view. This was accomplished by using as fiducial markers the fluorescent beads embedded in the sample. Using the *Multiview Reconstruction* plugin of *Fiji*^8^ these fiducial markers were identified in each view. Subsequently, each view was rotated to fit the reference frame of the first view. In Fig. 3 all views were registered to *View 1* and from here on it is referred to as Single View (SV) and is used as comparison with the MVI and MVDA images. In each view the direction of the z-stack is indicated with the z axis sign. The registered views were fused into one image (Fig. 3E). While in each individual view only a part of the sample could be seen, in the fused image clearly the entirety of the sample became visible with all the details from all the views. The fused image constitutes the MVI image. However, because of the elongated PSF on the z axis of each view, the MVI image appeared blurrier than each individual view. To remove this effect, deconvolution was applied. In this process, the PSF of each view, extracted from the fluorescent beads of each view, were used to perform the deconvolution using the *Multiview Reconstruction* plugin of *Fiji*. The result is presented in Fig. 3F. Clearly, contrast in the MVDA image is higher compared to the MVI image, while the entirety of the sample remains visible.

**Figure 4:**
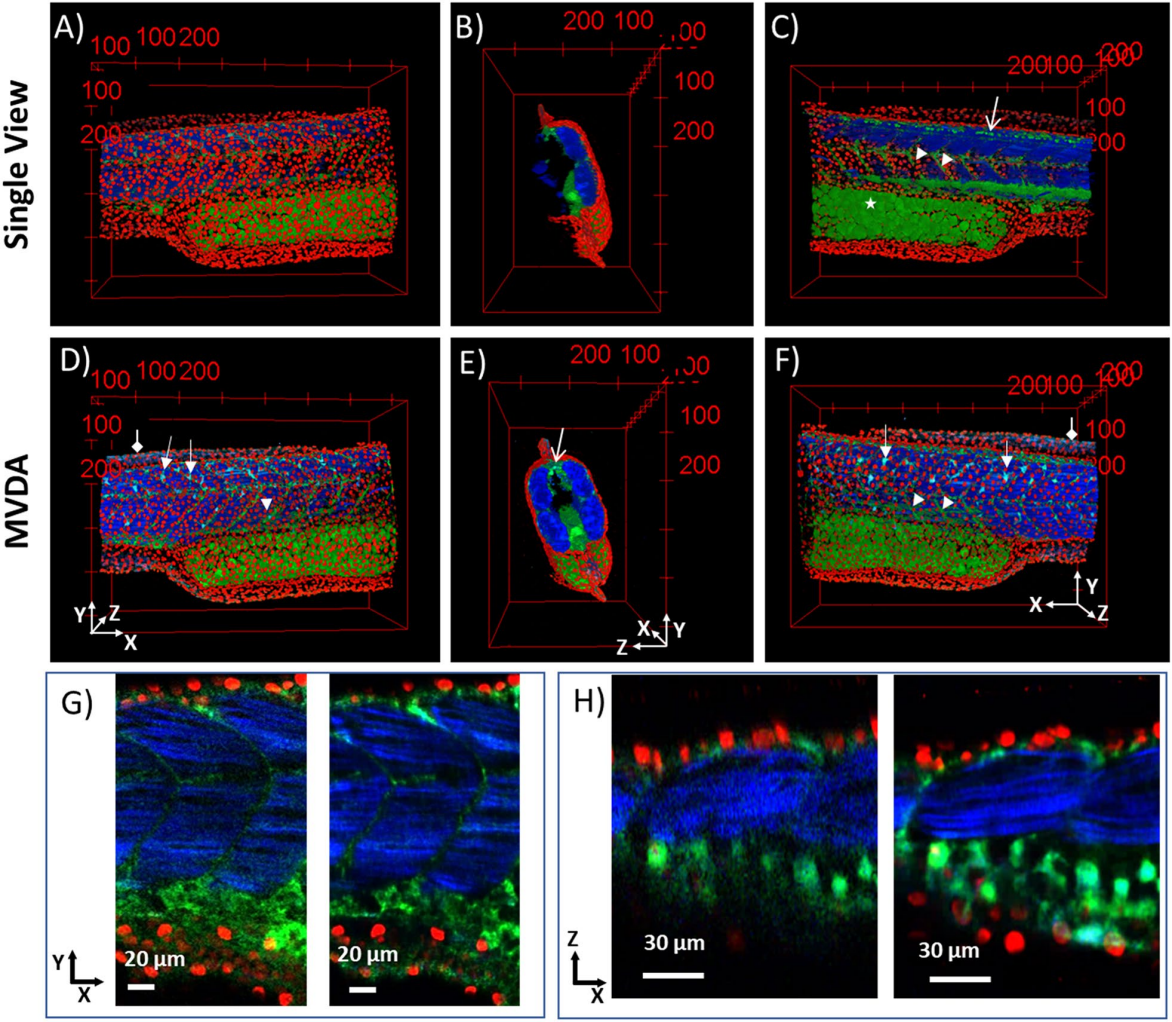
3D images of SV and MVDA. Nuclei in red, autofluorescence and sensory neurons (GFP) in green, muscle fibres and collagen in blue. (**A**) SV image, (**B**) SV image rotated 90° to the right. (**C**) SV rotated 180° compared to (**A**). (**D**) MVDA image with the same viewing perspective as A). MVDA image rotated 90° to the right compared to (**D**). (**F**) MVDA rotated 180° compared to (**D**). Arrow heads: myotomes, Diamond arrows collagen in the fins, Open arrows: sensory neurons, Arrows: dendritic like cells. (**G**) 2D images in the XY plane of SV left and MVDA right. (**H**) 2D images in the XZ plane of SV left and MVDA right. (Images were created with Fiji^26^).

In Fig. 4 the 3D reconstruction of the SV and MVDA images are presented. In Fig. 4A the SV is seen from the XY perspective. The outer structure of the fish is visible. Nuclei on the skin are seen in red, autofluorescence from the yolk sac and myotomes are seen in green, and muscles in blue. In the corresponding MVDA image (Fig. 4D) the same structures are visible, however they appear crisper. Nuclei appear smaller because they are less blurry with more well-defined borders. Also, lipids in the yolk sac and myotomes appear crisper because resolution is better, and noise is reduced. What becomes also better visible are cells near the skin surface, that were faintly visible on the SV (arrows). This is because structures with dim signal are enhanced in MVD. The nature of these cells is not known as there is no specific stain, but since they have a dendritic like structure, they could be cells of the immune system. Muscle fibres also look clearer as also collagen in the fins (diamond arrow) which is faintly visible in the SV.

In Fig. 4B and E the 3D images are rotated 90° to the right and the sagittal view (XZ) is visualized. In the SV image only part of the sample is visible since penetration depth was restricted (~ 120 μm). In the MVDA image all parts of the sample are visible with good contrast. From this viewing angle the GFP signal can be discerned. Spectrally, it is difficult to separate the GFP signal from autofluorescence but based on morphology^24,27^ it is possible to distinguish the GFP signal on the spinal cord, where bright dots from the nervous cell body are visible (open arrow). In the MVDA image the full structure of the sample is visible with equal contrast. Additionally, muscle fibres in both sides of the backbone and the skin cells surrounding the fish body are visible.

In Fig. 4C and F the 3D images are rotated additionally 90° to the left, so the other side of the sample is presented. In the SV image, since penetration depth was limited, only half of the sample is visible, and this allows a good view of internal structure where muscle fibres, myotomes, the yolk sac (star), sensory neurons (open arrow) and cell nuclei are visible. In the MVDA image similar to Fig. 4D all these structures appear crisper and better resolved.

In Fig. 4G and H magnified images from different parts of the sample are presented, in order to demonstrate the improvement in resolution. In Fig. 4G the SV and MVDA on the XY imaging plane are presented where the improvement in resolution in all channels is evident. In Fig. 4H the SV and MVDA on the XZ imaging plane is presented where the muscle fibrils are better discerned and autofluorescence structures and nuclei are crisper in the MVDA image. These images are presented with additional details in supplementary information Figures S1 and S2.

Conclusively in the MVDA image the entirety of the sample is visible with better resolution, and higher contrast. The improvement in resolution is further demonstrated in the supplementary figures (Figures S1, S2, and S3) where it is shown that nuclei are better resolved and, muscle fibres can be discerned in the XZ and YZ axis while this is not possible in the SV image. Noise is reduced which allows imaging of faint signal of autofluorescence structures to be visualized clearly. Moreover, the benefits of MVDA are demonstrated for both fluorescence and SHG signal, which can be a significant advantage when imaging unstained samples based only on the inherent signal. The improvement in contrast and resolution is demonstrated in 3D images in the Supplementary Video 1.

### Multiview deconvolution of collagen SHG with 4 views and 8 views

In the previous section, the benefits of 4-view MVDA on axial resolution improvement and visualization of the entirety of the sample in fluorescence and SHG contrast were demonstrated. In this section, further improvement in resolution in SHG contrast by increasing the number of views was investigated. For this purpose, a rat-tail tendon containing mainly parallel organized collagen fibres was used.

**Figure 5:**
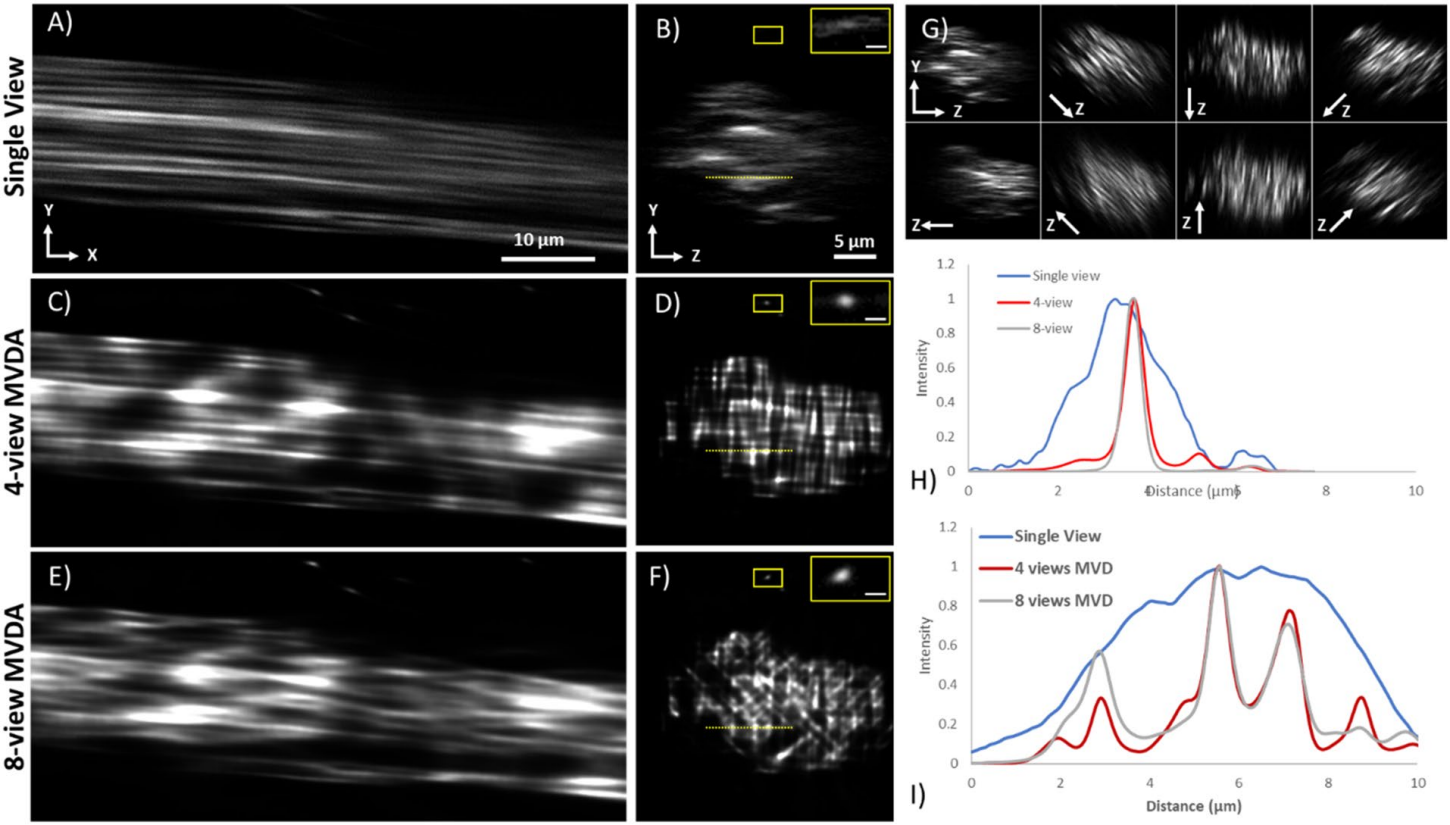
Rat-tail tendon imaging based on SHG. Single view (**A**) XY plane, (**B**) YZ plane (sagittal section). 4-view Multiview deconvolution (**C**) XY plane (**D**) YZ plane (sagittal section). 8-view Multiview deconvolution (**E**) XY plane (**F**) YZ plane (sagittal section). (**G**) YZ planes (sagittal sections) of all individual registered views. The direction of the Z-axis is indicated in each view. (**H**) Normalized intensity plot profiles of the fibre on the insets of (**B**), (**D**), and (**F**) contrast has been increased for visualization. (**I**) Normalized intensity plot profiles of the dotted lines in (**B**), (**D**), and (**F**). More individual fibres are revealed in the profiles of 4-view MVDA and 8-view MVDA. Scale bar on the inset of (**B**), (**D**), (**F**) 1 μm. (Images were created with Fiji^26^).

In Fig. 5 the SV (Fig. 5A, B), the 4-view MVDA—90° rotation step—(Fig. 5C, D), and the 8-view MVDA—45° rotation step—(Fig. 5E, F) of a rat-tail tendon based on SHG signal are presented. Rotation axis was on the x-axis as indicated in Fig. 5. To exploit the full resolution capabilities of the microscope, images were acquired with high sampling density—pixel size smaller than microscope resolution. Acquisition time was in average 3 min for each view with a FOV of 105 × 105 μm^2^ and voxel size of 103 × 103 × 494 nm. The individual registered views are presented in Fig. 5G.

In the lateral XY plane of the SV image (Fig. 5A), collagen fibres appear well defined and continuous. In the 4-view (Fig. 5C), and the 8-view (Fig. 5E) MVDA images, collagen fibrils do not appear as continuous as in the SV. This impression is created because fibrils are three-dimensional, and their axis does not exactly coincide with the XY imaging plane. In reality, the fibrils cross the imaging plane, but because of the poor axial resolution in the SV they appear to be along the XY imaging level. On the other hand, in the MVDA images, fibrils appear to come in and out of the imaging plane, therefore they do not appear as continuous as in the single view.

In the sagittal (YZ plane) sections of the SV (Fig. 5B), the SHG signal from fibrils overlap creating blurred elongated structures where individual fibrils are not clearly resolvable. In the MVDA sections (Fig. 5D and F) individual fibres become visible. In the 4-view MVDA, a striping effect is visible. This striping has the direction of the z-axis of each individual view and is due to the incomplete removal of the PSF elongation after deconvolution caused by strong local constructive interference effects observed in such tissues^28,29^. The addition of more views minimizes this effect, and individual fibrils become better visible (Fig. 5F). On the magnified inset of Fig. 5B, D and E a single fibre which is isolated from other fibres is analysed to demonstrate resolution improvement. While in the SV image contrast is low, in the MVDA images contrast is much higher. In the SV image the fibre appears elongated in the direction of the Z-axis, in the MVDA images the fibre appears rounder. The corresponding plot profile is presented in Fig. 5H. The apparent diameter of the fibre in the Z-axis in the SV is 2112 ± 152 nm, in the 4-view MVDA is 520 ± 42, and 461 ± 35 in the 8-view MVDA.

In Fig. 5I comparison of the normalized intensity profile of the dotted line in Fig. 5B, D and F are presented. In the SV only a single structure is visible, while in the MVDA images more peaks are present indicating the presence of more fibrils.

**Figure 6:**
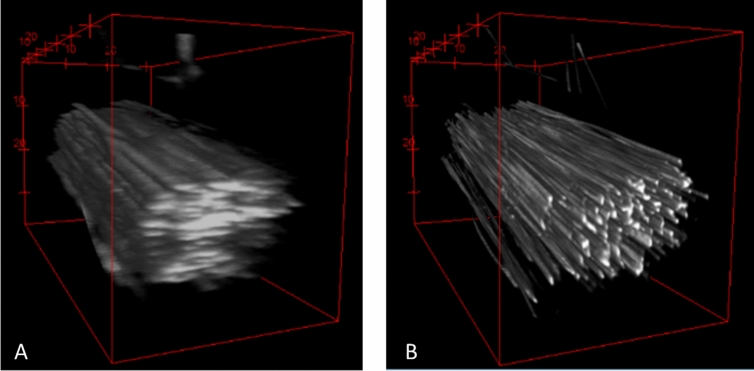
3D rendering of rat-tail tendon. (**A**) Single view. (**B**) 8-view MVD. In the MVD single fibrils are clearly visible. (Images were created with Fiji^26^).

To better demonstrate the advantages of MVDA the 3D images are presented in Fig. 6. The SV image appears very blurry, especially in the Z-axis. In the 8-view MVDA image many more fibrils can be resolved and gives a clearer visualization of the tendon. Quantification of the number of fibrils based on the 8-view MVDA can yield more accurate results. The animation of the 3D reconstruction of Fig. 5 are provided in Supplementary Video 2.

### MVDA on Zebrafish heart

**Figure 7:**
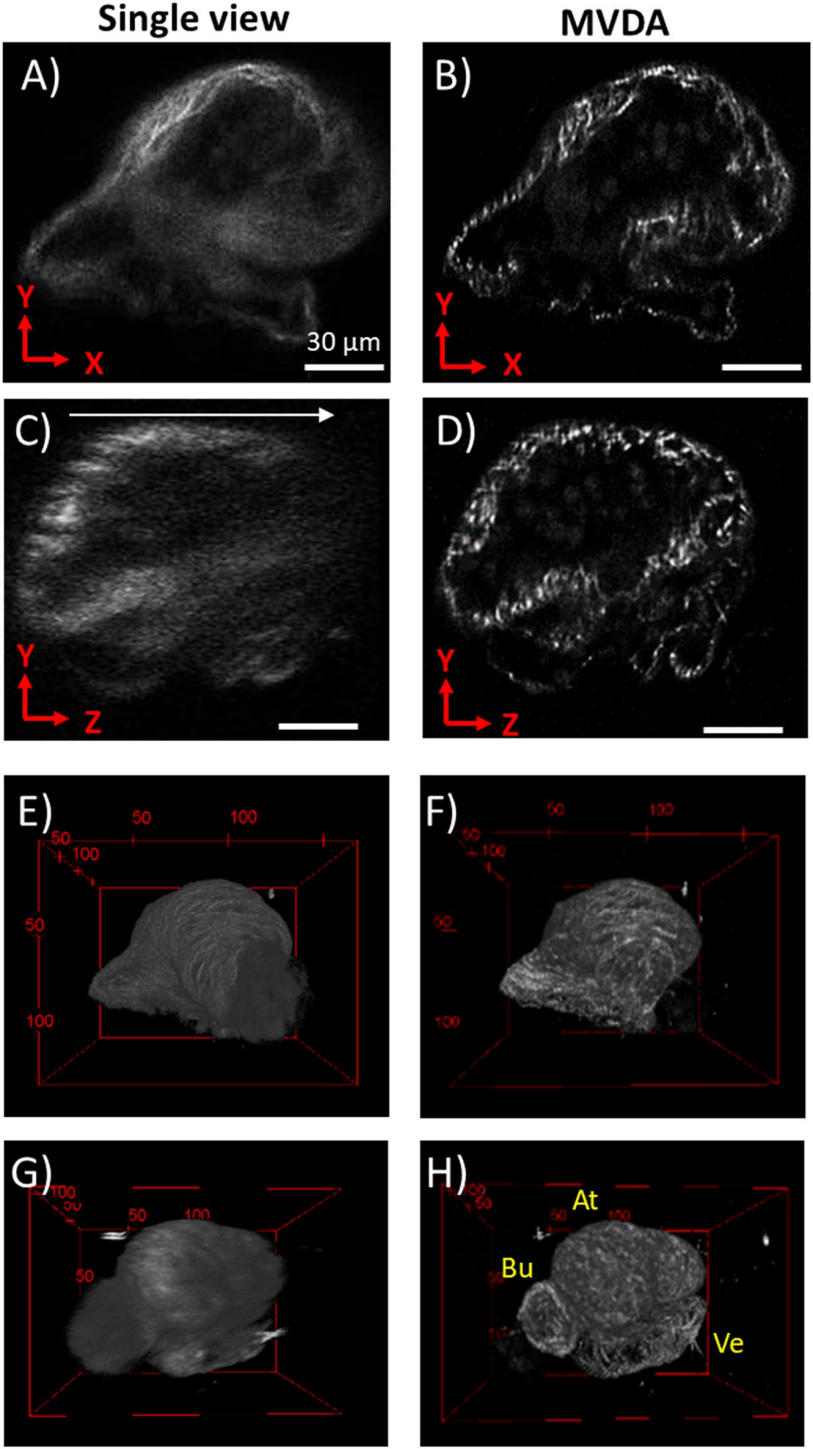
SHG images of 3-dpf zebrafish heart. (**A**) Single view YX imaging plane. (**B**) MVDA YX imaging plane. (**C**) SV sagittal section (YZ plane), arrow indicates direction of z-stack. (**D**) MVDA sagittal section. Scale bar: 30 μm. (**E**–**H**) 3D reconstructions of the zebrafish heart. (**E**), and (**G**) SV, (**F**) and (**H**) MVDA. (**F**) and (**H**) rotated 90° compared to (**E**) and (**G**) respectively. At: Atrium, Ve: Ventricle, Bu: Bulbus arteriosus. (Images were created with Fiji^26^).

In the previous section the strong SHG signal from collagen was imaged. In this section the advantages of using the MVDA method to image the faint SHG signal from myosin of a 3-dpf zebrafish heart was explored. The signal from myosin fibrils of the developing heart was low, which resulted to very low SNR and noisy images (Fig. 7C). For this reason, 8-view MVDA setting to capture most of the detail of this faint signal was used. To avoid sample photodamage a relatively large voxel size was used (206 × 206 × 1280 nm). Rotation axis was on the x-axis as indicated in Fig. 7. To increase the detected SHG, laser power was increased compared to normal fluorescence imaging. This caused some autofluorescence cross talk in the SHG channel, however this signal was well below the SHG levels. The results are given in Fig. 7. In the SV image, in the XY imaging plane, (Fig. 7A) some myofibrils are clearly visible; however, the image has low contrast and structures inside the heart are not well defined. In the corresponding MVDA image (Fig. 7B) myofibrils appear well defined and with high contrast. In the sagittal (YZ) plane, the SV image (Fig. 7C) signal in the upper layers of the image is high, while it is reduced at deeper layers (arrow indicates the direction of the z-stack). Also, the structures visible in the upper layers are blurry because of the low resolution in the axial dimension. On the corresponding MVDA image (Fig. 7D) noise is reduced and structures are visible with comparable contrast in all depths of the sample. Individual fibrils are clearly visible throughout the sample with high contrast, therefore MVDA provided a detailed image of a sample with faint signal, that was not attainable with other means.

The benefit of performing MVDA is illustrated even better by the 3D reconstruction images (Fig. 7E–H). In the MVDA images (Fig. 7F and H) the entirety of the heart is visible, as are also individual cardiac myofibrils. In contrast, the SV images (Fig. 7E and G) are very blurry and contrast at the deeper layers is low. The Atrium, Ventricle, and Bulbus arteriosus of the heart are clearly visible in Fig. 7H. The animation of the 3D reconstructions of Fig. 7 are provided in Supplementary Video 3. Interestingly, the structure of the developing heart is very different to that of a mature heart. Cardiac muscle fibres are not so dense, and appear to be crossing in between, but do not have a precise directional order as is seen in mature hearts. In the developed heart the entire heart wall is expected to be covered by muscle fibres. Imaging of the intrinsic signal of the heart is particularly interesting when investigating cardiomyocyte morphology in cardiomyopathies and genetic diseases such as laminopathies, especially in developing model organisms such as the zebrafish embryo.

## Discussion

In this study the feasibility and benefits of applying MVDA on conventional MPM was demonstrated. This was accomplished by developing a custom rotation chamber where the sample could be mounted and rotated on a horizontal configuration. Registration, fusion, and deconvolution of the MVDA image was performed based on the general public licence plugin *Multiview Reconstruction*^8^ of *Fiji*. MVDA imaging, clearly demonstrated improvement in resolution as well as imaging the entirety of the sample. Additionally, low signal structures became better visible in the MVDA image (Figures S1, S2, and 7). Improvement in resolution was demonstrated in both fluorescence and SHG signal. This method could be applicable to small organs or tissue sections, bigger cleared organs or tissue sections, and small organisms such as zebrafish embryos.

The PSF of SHG depends strongly on the local sample structure making its definitions challenging, and for this reason deconvolution applications have been limited^30^. SHG is a coherent phenomenon, emission is not isotropic as fluorescence, and is mainly in the direction of the excitation beam. SHG signal depends on the organization of the emitters (the harmonophores), the intensity and polarization of the excitation light, and the orientation of the harmonophores relative to the excitation beam^19,31^. As a consequence, resolution in SHG imaging cannot be described accurately by a single PSF. However, since the PSF of an imaging system is the product of the excitation PSF and the emission PSF, it was chosen to use the same PSF for SHG as for fluorescence, since they have common excitation PSF. We chose to name our method as Multiview deconvolution-approximation to differentiate it from classical deconvolution where a spatially invariant PSF can be defined accurately. SHG is a coherent phenomenon and as such SHG images should be rather viewed as coherence patterns and not as harmonophore density. However, in literature, SHG is a well-established contrast method for imaging^32^ and is used for visualization and quantifying collagen^33^ or myosin content, even though those images may contain illusionary artifacts^34–36^. The advantage of visualizing those structures at all, is negating the disadvantage of artifact presence. We used this approximation to explore the benefits and challenges of Multiview imaging of coherent signals and asses its feasibility. In this case it was shown that for SHG, increased number of views can provide better results. Based on this approximation a significant improvement in resolution was demonstrated (Fig. 5, SV: 2112 nm, MVDA: 461 nm). This size corresponds well with the thickness of rat tail tendon fibril (100–500 nm) as characterized with scanning electron microscopy^37^.

In the tissue level where multiple fibrils in collagen, and multiple myofibrils in muscle, bundle together in fibres, it is difficult to separate them in SV. In MVDA, these structures were clearly discerned in the axial dimension. In the SV, constructive interference between emitters can enhance the signal between fibrils making their visual separation impossible. An example of such interference phenomena is the Vernier like artifacts reported for muscle fibres^34,35^ and the elongated collagen fibrils seen in the Z-axis (Figs. 5, 6). Moreover, it has been shown that SHG depends on the orientation of collagen fibrils ^36,38^.These interference phenomena are view specific, and usually are present in one view but not the others. When the MVDA image is formed the view specific interference artifacts tend to be supressesed by the inherent signal of the fibril, which is present in every view, and therefore lead to a clearer image. In MVDA such artifacts are greatly suppressed and allow for clear visualization of the myofibrils (Figures S1 and S2). Such interference phenomena are also present in the 4-view MVDA (striping effect), therefore increasing the number of views significantly supressed them, as was shown in the 8-views MVDA (Fig. 5). Interferometric second harmonic generation microscopy^28,29^ has been proposed to eliminate interference artifacts with very promising results. Indeed, such artifacts are completely suppressed offering a clear view of the inherent SHG signal, although this technique is very sensitive, it is limited to thin tissue sections.

Regarding the number of views in MVD, in this study only 4 and 8 views were explored, but any other combination is possible. The number of views depends on the resolution improvement required but also on the sample optical properties. In theory close to isotropic resolution can be achieved with only 4 views but for thicker samples not all details are captured equally in every view. In a study based on SHG signal MVI, it was shown that a intertwining collagen fibre was visualized more clearly using 10 views^17^, whereas for LSFM MVI up to 24 views might be necessary to capture all the details of a large sample and to overcome local distortion due to scattering and refraction within the sample^39^. Recently it was shown that for samples not clearly visible in all views selecting pixels rich in information can provide better results than blindly incorporating all views in the MVD^39^. Therefore, such selection strategies should be considered for future applications. Moreover, big data size and processing speed, are increasingly limiting in Multiview imaging and efforts to improve speed based on graphics cards processing and use of deep leaning methods to improve deconvolution have been recently demonstrated with promising results^40^.

MPM is a point scanning technique and as such is slower compared to the widefield detection based LSFM. In the cases presented here, acquisition time was ~ 25 min which can be limiting when higher temporal resolution is required. The lack of speed however can be compensated by several unique advantages. Firstly, imaging of direct SHG signal is possible with MPM. In LSFM, because of the perpendicular geometry of excitation and detection only scattered SHG signal can be detected, so forward emitted SHG from muscle would not be detectable even if two-photon excitation was used. For collagen SHG, LSFM has been demonstrated only on backscattered signal^41^, which results to lower contrast images compared to conventional MPM. Secondly, in MPM, since there is a single objective for excitation and detection, the field of view does not have to be sacrificed for resolution. In typical LSFM geometries, improvement in axial resolution is at the expense of Field of View^42^, which can be limited to 20 × 20 μm ^43^. Thirdly and finally, spectral imaging can be more efficient. MPM can excite and detect different fluorophores simultaneously therefore reducing the acquisition time and offering more options for the number of stains used. In the current discussion novel LSFM techniques such as Lattice light sheet^42,43^ or Super-resolution^44^ are not considered. It would be wiser to compare such techniques with MVDA performed under STED excitation, which is in our immediate future plans. The MVDA approach described here, apart from MPM, could be also applied to any conventional laser scanning technique such as confocal microscopy or their super resolution equivalents. The only additional requirement would be a suitable rotation chamber and powerful enough computer for MVDA. Microscope modifications would not be required.

So far, no modification of the microscope has been proposed, however modifications to increase speed could be beneficial. In the community of LSFM simultaneous MVI has been demonstrated. For example multiple objectives positioned in opposing directions and light-sheet excitation and detection of different views performed simultaneously has been demonstrated^11,45^. With a similar configuration implemented for MPM, MVI acquisition would require the same time as a single view. However, this would also add limitations to sample size and positioning of the objectives. Another possibility to increase the acquisition speed would be to use multi point excitation^46,47^. In this case, a single beam can be split up to 64 beams that simultaneously scan different parts of the field of view, resulting to up to 64 times faster scanning of a single image. This would significantly increase the acquisition time of each individual view and rotation of the sample and size would not be restricted. However, it should be taken into consideration that axial resolution and penetration depth of multibeam excitation can be inferior to that of the single beam^48^. Nonetheless, the speed increase could be a significant advantage for samples up to 200 μm thick, such as zebrafish larvae, and could be a promising alternative. Therefore, even though MPM MVDA is still relatively slow as demonstrated in this study, it can potentially become much faster through microscope modifications, maybe even fast enough to image dynamic phenomena with temporal resolution of some seconds.

## Supplementary Information

Supplementary Information.

Supplementary Information.

Supplementary Information.

Supplementary Information.

Supplementary Information.

Supplementary Information.
