# Supplementary material for: Multiview deconvolution approximation multiphoton microscopy of tissues and zebrafish larvae
Source: Sci Rep. 2021 May 12;11:10160. doi: 10.1038/s41598-021-89566-w (PMC8115086; doi:10.1038/s41598-021-89566-w)
Supplement: Supplementary file 2 — Supplementary Information. [file 41598_2021_89566_MOESM2_ESM.docx]

Supplementary Information

Multiview deconvolution approximation multiphoton microscopy of tissues and zebrafish larvae

Dimitrios Kapsokalyvas^1,2^, Rodrigo Rosas^1^, Rob W.A. Janssen^1^, Jo M Vanoevelen^1^, Miranda Nabben^1^, Martin Strauch^3^, Dorit Merhof^3^, Marc AMJ van Zandvoort^1,2^

^1^Department of Genetics & Cell Biology, Faculty of Health, Medicine and Life Sciences (FHML), Maastricht University, Maastricht, the Netherlands.

^2^Institute for Molecular Cardiovascular Research (IMCAR), University Hospital RWTH Aachen University, Aachen, Germany.

^3^Institute of Imaging & Computer Vision, RWTH Aachen University, Aachen, Germany

**Multiview on Zebrafish larva**

In Figure S1 the XY plane images of the SV, and the MVDA images are presented. The SV image is noisier and not all parts of the embryo appear with high contrast. This is especially evident for the green structure in the lower part of the image. This structure is formed by the autofluorescence of lipids and appears dim in the SV image, while it is well visible in the MVDA image. The MVDA is less noisy, structures appear crisper, and all parts of the embryo are well visible. In the red channel, nuclei appear with similar dimensions in the XY plane (Figure S1E), while in the green channel the myotomes are visible with less noise in the MVDA image compared to the SV image (Figure S1F). In the blue channel (Figure S1F), individual muscle fibres (SHG) in the MVDA image are visible with higher contrast compared to the SV image, although the muscle striation is not as clear as in the SV. This can be ascribed to the better lateral resolution in SV images.


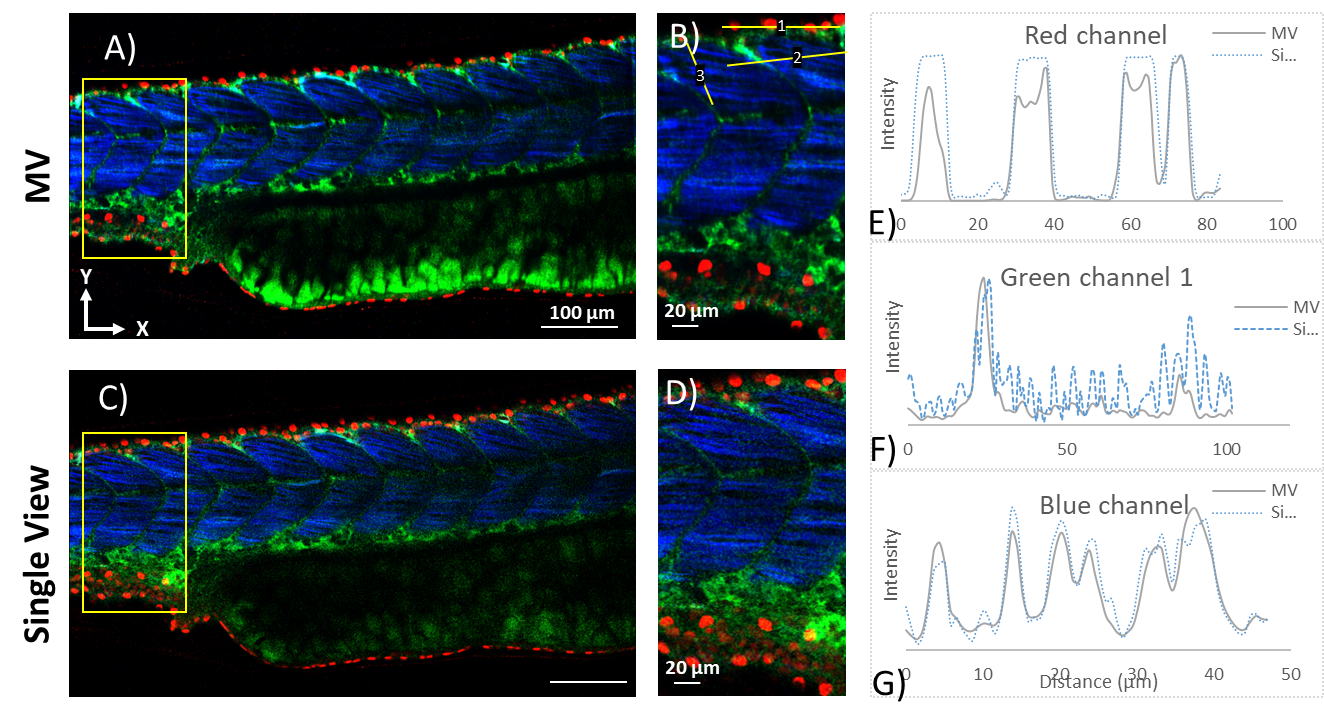


**Figure S1:** Images of MV and Single view in the XY plane. Red channel: nuclei, Green channel: Autofluorescence and GFP, Blue channel: SHG from muscle in the main body of the fish. A) MV image, B) Magnified image of the inset in A). C) Single view, D) Magnified image of the inset in C). Normalized intensity plot profiles for the lines indicated in B) for each corresponding channel. E) Red channel (line 1), F) Green channel (line 2), and G) Blue channel (line 3). (Images were created with Fiji^1^).


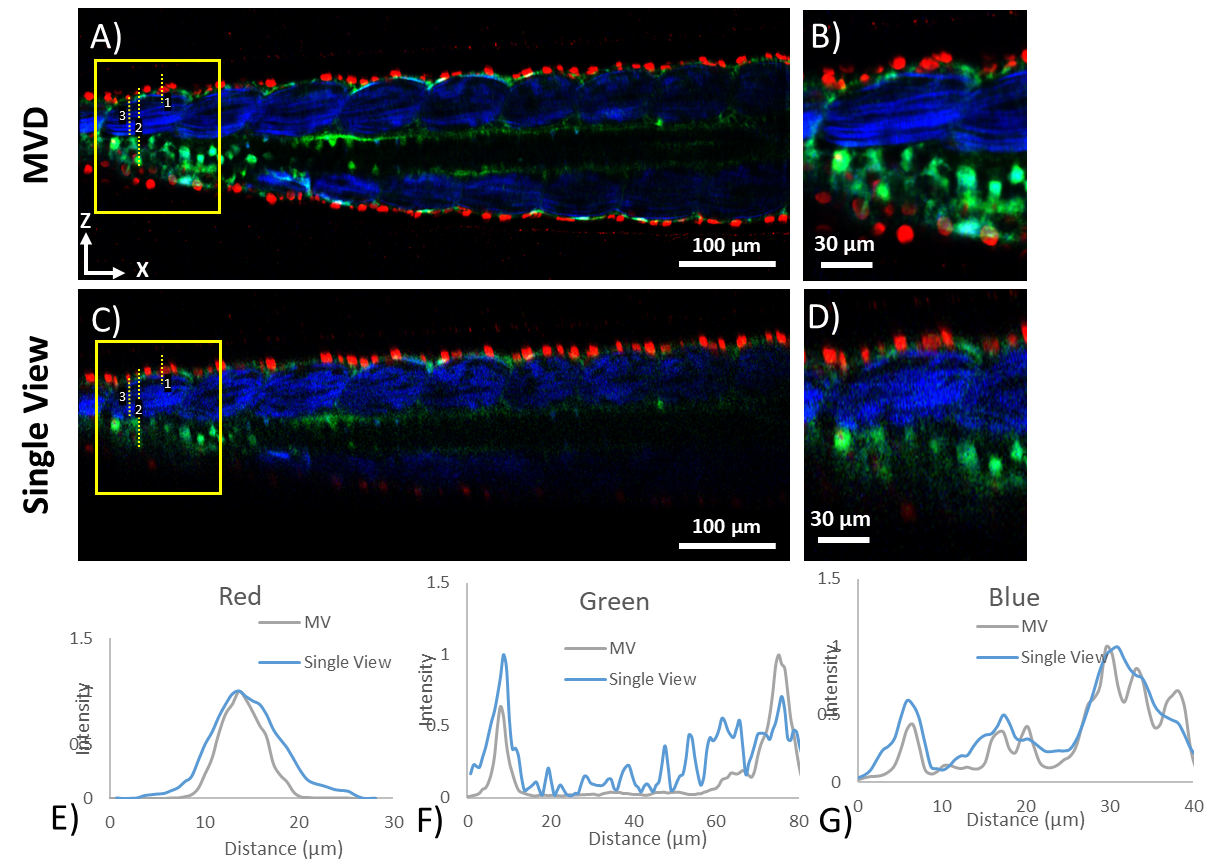


**Figure S2:** Longitudinal sections of MV and Single View. Image coding same as Figure S1. A) MV image, B) Magnified image of the frames in A). C) Single View, D) Magnified image of the inset in C). Normalized intensity plot profiles for E) line 1, red channel, F) line 2, green channel, and G) line 3, blue channel. Grey line is MV and light blue line is SV. (Images were created with Fiji^1^).

In Figure S2, the longitudinal XZ sections of the sample are presented. In Figures S2A and S2C the full field of view is presented. In the MVDA images (Figure S2A) the entirety of the sample is visible, while part of that is not visible in the SV images (Figure S2B). A magnified view of the frames in Figures S2A and S2C are presented in Figures S2B and S2D, respectively. In the MVDA, noise is reduced, and fluorescent features are visible with higher contrast. In Figure S2E- S2G the intensity plot profile of the yellow numbered lines in Figures S2A and the corresponding regions of S2C are presented. In Figure S2G, nuclear size is smaller in the MVDA indicating improvement in resolution. In Figure S2F, the profile of the MVDA image is less noisy compared to the SV and the fluorescent structures appear smaller, which is a further indication of resolution improvement. Better contrast of the MVDA image is also evident in the green channel of Figure S2B where sensory neurons expressing GFP are better visible compared to the SV (Figure S2D). In the blue channel (SHG), individual muscle fibres become more clearly visible in the MVDA image compared to the SV. The intensity plot of line 3 in Figure S2A is presented in Figure S2G. The MVDA has more peaks compared to the SV, which means that more individual fibres were resolved. While in the SV image striation of the muscle is better resolved than in the MVDA image, due to better resolution in the X axis, the borders between different fibres are not resolvable, a clear indication of resolution improvement in the axial dimension in the MVDA image. Clearly, new information is available in the MVDA image.


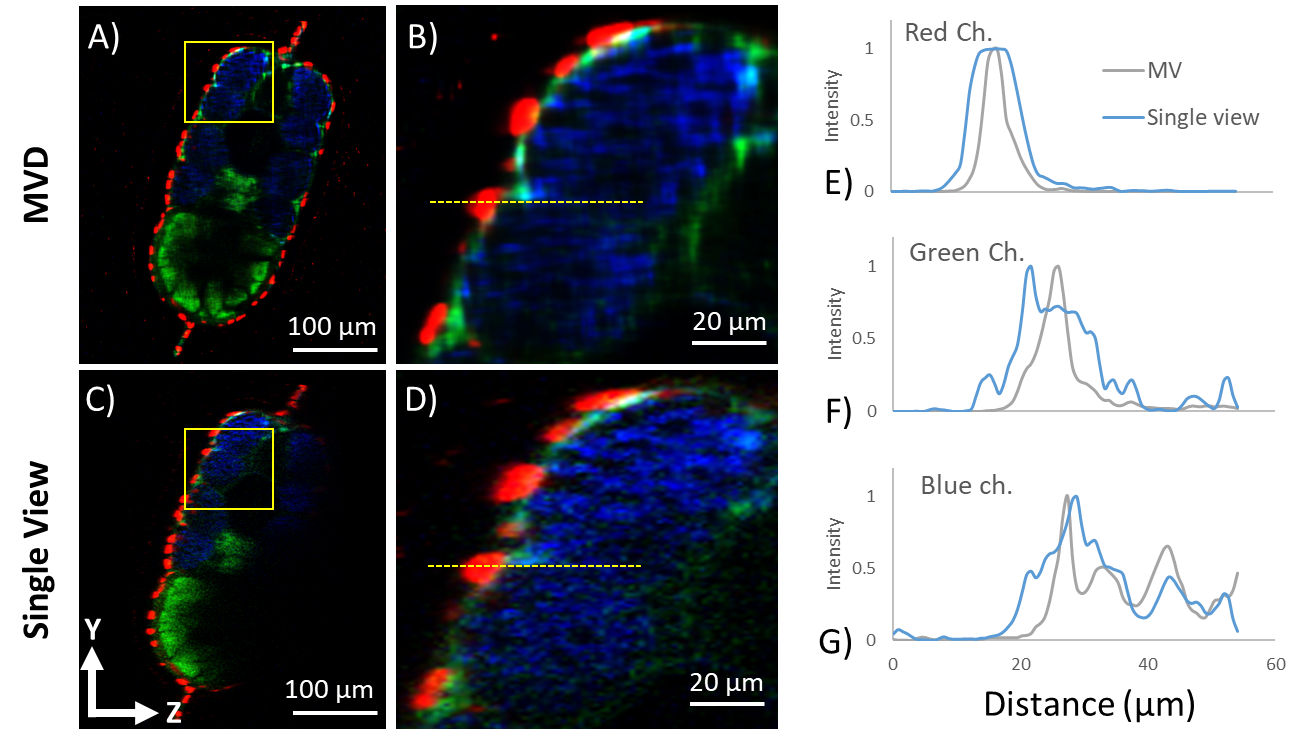


**Figure S3:** Sagittal sections of the MV and Single view images of a 3dpf zebrafish sample. A), B) MV, C),D) Single View. B) and D) are magnifications of the insets in A) and C) correspondingly. E), F) and G) Intensity plots of the red, green and blue channels of the yellow dotted line in B) and D). Gray line is MV and light blue line is Single View. (Images were created with Fiji^1^).

In Figure S3, the sagittal YZ sections of the MVDA and SV are presented. As expected, the entirety of the image is visible in the MVDA (Figure S3A) image, contrary to the SV (Figure S3C). Resolution improvement is better visible in the magnified views of the insets in Figures S3B, and S3D. Nuclei and autofluorescent structures appear better defined. This becomes apparent also in the graphs of Figures S3E, S3F, and S3G, which are the intensity plot profiles of the dotted line in Figure S3B and S3D. Nuclei are smaller and autofluorescent structures appear thinner and less noisy, as it was also observed in Figures S2E, and S2F. Muscle fibres based on SHG signal in the blue channel are better discerned compared to the SV.

In conclusion, careful examination of Figures S1,S2, and S3 clearly shows that all sides of the sample are well and clearly visualized in MVDA, leading to an increase of the overall 3D field of view. Improvement in the resolution of fluorescent structures is also clearly visible in the images, but is also demonstrated in the plot profile graphs of Figures S2, and S3. Resolution improvement is also demonstrated for SHG signal as individual muscle fibres can be discerned in the lateral but also axial dimension.

References

(1) Schindelin, J.; Arganda-Carreras, I.; Frise, E.; Kaynig, V.; Longair, M.; Pietzsch, T.; Preibisch, S.; Rueden, C.; Saalfeld, S.; Schmid, B.; et al. Fiji: An Open-Source Platform for Biological-Image Analysis. *Nat. Methods* **2012**, *9* (7), 676–682.
