# Supplementary material for: Multiview deconvolution approximation multiphoton microscopy of tissues and zebrafish larvae
Source: Sci Rep. 2021 May 12;11:10160. doi: 10.1038/s41598-021-89566-w (PMC8115086; doi:10.1038/s41598-021-89566-w)
Supplement: Supplementary file 6 — Supplementary Information. [file 41598_2021_89566_MOESM6_ESM.docx]

**Supplementary media**

Supplementary video 1: The 3D reconstruction of the zebrafish image. Left: SV. Right: MVD. In the MVDA the entirety of the sample is visible, and contrast is enhanced. Colour coding, Red: Nuclei, Green: Autofluorescence and GFP, Blue: SHG from muscle in the body of the fish and collagen in the fins.

Supplementary video 2: The 3D reconstruction of the collagen fibre’s SHG signal. Left: SV. Right: MVDA. In the MVDA image single fibrils are clearly discerned.

Supplementary video 3: The 3D reconstruction of the zebrafish’s heart SHG signal. fibre. Right: SV. Left: MVDA. In the MVD myofibrils become visible, whereas in the SV the whole structure appears blurry.

[Supplementary file 1: CAD MV rotation chamber.obj](https://submission.nature.com/submission/4878a1dc-d493-4dfd-b403-1eff9468b618/file/d5fa0c70-f6dd-40b2-9f60-81bf9b9292f2) (CAD file of the rotation chamber).
